# Automated analysis of spontaneous eye blinking in patients with acute facial palsy or facial synkinesis

**DOI:** 10.1038/s41598-024-68707-x

**Published:** 2024-07-31

**Authors:** Lukas Schuhmann, Tim Büchner, Martin Heinrich, Gerd Fabian Volk, Joachim Denzler, Orlando Guntinas-Lichius

**Affiliations:** 1 Department of Otorhinolaryngology, Jena University Hospital, Friedrich Schiller University Jena, Am Klinikum 1, 07747 Jena, Germany; 2 Computer Vision Group, Friedrich Schiller University Jena, Jena, Germany; 3 Facial-Nerve-Center Jena, Jena University Hospital, Jena, Germany; 4 Center for Rare Diseases, Jena University Hospital, Jena, Germany

**Keywords:** Neurology, Eye diseases

## Abstract

Although patients with facial palsy often complain of disturbed eye blinking which may lead to visual impairment, a blinking analysis is not part of routine grading of facial palsy. Twenty minutes of spontaneous eye blinking at rest of 30 patients with facial palsy (6 with acute palsy; 24 patients with facial synkinesis; median age: 58 years, 67% female), and 30 matched healthy probands (median age: 57 years; 67% female) was smart phone video recorded. A custom computer program automatically extracted eye measures and determined the eye closure rate (eye aspect ratio [EAR]), blink frequency, and blink duration. Facial Clinimetric Evaluation (FaCE), Facial Disability Index (FDI) were assessed as patient-reported outcome measures. The minimal EAR, i.e., minimal visible eye surface during blinking, was significantly higher on the paretic side in patients with acute facial palsy than in patients with synkinesis or in healthy controls. The blinking frequency on the affected side was significantly lower in both patient groups compared to healthy controls. Vice versa, blink duration was longer in both patient groups. There was no clear correlation between the blinking values and FaCE and FDI. Blinking parameters are easy to estimate automatically and add a functionally important parameter to facial grading.

## Introduction

Eye blinking and the underlying blink reflex are an important functions to protect the eye^1^. The blinks are mainly realized spontaneously, but also voluntarily. Most important is the facial nerve mediated contraction of the orbicularis oculi muscle^2^. Patients with acute facial paralysis are unable to blink on the affected side. This leads to eye irritation and impairment of the tearing function^3^. All types of blinking are impaired in the acute phase of the palsy^1^. Depending on the etiology, not all patients recover completely, but can develop a postparalytic facial nerve syndrome with synkinesis^4^. It seems that synkinesis leads to less effective eyelid movement during blinking^5^. Patients with acute or chronic facial palsy report a severely decreased quality of life^6^. Facial-specific patient-reported outcome measures (PROMs) show that the disturbed protection function has a major impact on quality of life^7,8^. Nevertheless, blinking function and quality life were not yet directly compared. Moreover, blinking was traditionally and objectively measured by magnetic search coils on the eyelids and electromyography (EMG) of the orbicularis oris muscle^1^. Nowadays, manifold approaches are used for automated eye blink detection, for instance to detect car driver drowsiness^9,10^. There are first attempts to use automated image analysis for routine grading of patients with facial palsy^11,12^, and also to automatically extract eye function features out of videos of patients with facial palsy^13^.

Therefore, we developed a tool box for automated blinking analysis for patients with facial motor diseases using automated image analysis algorithms^14^. Herein, the first clinical application, i.e. a detailed analysis of spontaneous blinking in patients with acute facial palsy and patients with postparalytic facial nerve syndrome with synkinesis in comparison to healthy controls is presented. The main objective was to establish the tool box for use in clinical routine and to show the feasibility to measure objectively spontaneous blinking parameters. Secondary objectives were (1) to objectify impaired blinking in patients with facial palsy, and (2) to correlate this impairment with quality of life measures. We hypothesized that blinking remains impaired in patients with facial synkinesis and that such an impairment reduced the quality of life.

## Materials and methods

### Patients with facial palsy and healthy controls

The patient group consisted of 20 women and 10 men (age range: 22–81 years). The patients for this prospective observational study were recruited from the Facial-Nerve-Center Jena, Jena University Hospital, Germany during their treatment outside of this study, i.e. the characteristics of the facial palsy were extensively known^6^. The patients had to be adult (≥ 18 years of age), had an acute facial palsy (onset ≤ 7 days), or a postparalytic facial nerve syndrome with synkinesis (confirmed by EMG^4^). The gender and age matched healthy control group also consisted of 20 women and 10 men (age range: 22–82 years). As inclusion criterion, the participants had to be healthy. Subjects with a history of any neurological disease including facial palsy and diseases of the eye, or an active neurological disease as well as a history of facial surgery or previous eyelid surgery were excluded.

All experimental procedures with human subjects followed the institutional research committee's ethical standards and the 1964 Helsinki Declaration and its later amendments. The ethics committee of the Jena University Hospital approved the study (No. 2021-2199_1-BO). All participants gave written informed consent to participate in the study. Informed consent has also been obtained to publish the facial images in a publication.

### Facial grading and quality of life assessment

Grading was performed by House-Brackmann grading scale and by the Sunnybrook Facial Grading System (SFGS)^15–17^. The House-Brackmann grading scale is a six step scale from grade I (normal function) to grade VI (complete paralysis). The SFGS is a regional weighted system that rates three subscores: resting symmetry, the degree of voluntary facial muscle movement, involuntary muscle contraction (synkinesis). The three subscores are used to calculate a composite score (0 = total paralysis; 100 = normal function). The validated German versions of two patient-reported outcome measures (PROMs), the Facial Clinimetric Evaluation (FaCE) scale and the Facial Disability Index (FDI) were used^8,18–20^. The FDI questionnaire comprises 10 Likert-type questions, divided into two domains, and includes physical function and social/wellbeing function. The physical function scale is scored from − 25 (worst) to 100 (best). The social/well-being function scores range from 0 (worst) to 100 (best). Both FDI scales are summed to a FDI total score. The FaCE has six independent domains: social function, facial movement, facial comfort, oral function, eye comfort, lacrimal control, and a total score incorporating all domains. Each FaCE score ranges from 0 (worst) to 100 (best).

### Standardized video recordings

The videos were taken in the same examination room of the department with standard neon ceiling lightening. The participant sat in front of a standard computer screen (full HD-LED, 1920 × 1080 pixel, 58 cm display) at a distance of 50 cm to the eyes of the participant. The patient’s monitor was framed from below with LED-light panels (4 × 40 cm, 15 W/750 lm, Müller-Licht, Lilienthal, Germany). The videos were taken with a smartphone at 240 frames per second (iPhone 8, Apple), Cupertino, California). The smartphone was installed in the midline of the computer screen below the computer screen using a smartphone tripod. The distance between the camera and the eyes was 45 cm. The head was not fixed. The complete head was always visible in the camera cutout. The setting is shown in in Supplement Fig. 1. The participants were instructed about the procedure and watched all the same 20-min passage of an animal and nature film (Name: “Abenteuer Erde: Sommerwelten”, producer Marco Polo Film AG, 2019, Westdeutscher Rundfunk, Cologne, Germany). A neutral passage of the movie was selected (factual presentation, not humorous or dramatic). The recordings were stored in .mov image format.

### Automated blinking analyses

**Figure 1:**
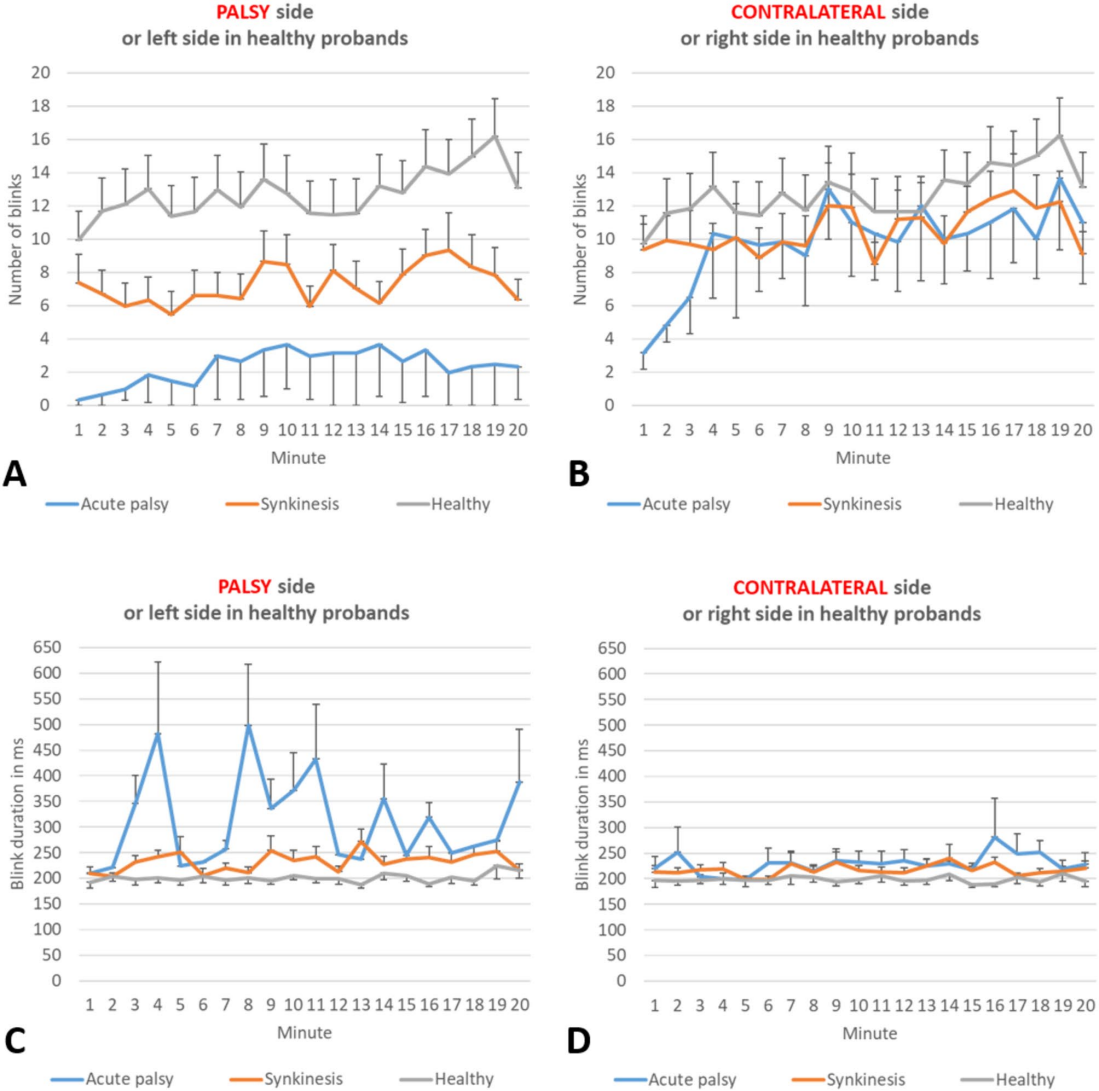
Explanation of the eye aspect ratio (EAR). EAR describes the ratio between the vertical and horizontal distance between the automatically detected landmarks. The formula for the calculation based on the landmarks is shown in the figure. EAR is characterizing the eye openness in each frame and invariant to the distance of the eye to the camera. The EAR is getting close to zero when closing the eye in a healthy person. The dynamics from normal openness to minimal openness during eye closure over time is shown form left to the right.

The Jena Facial Palsy Toolbox (JeFaPaTo) was used for the analyses^14^. In brief, JeFaPoTo performs first an automatic face detection in the imported video. Then, using the mediapipe library 468 facial landmarks and 52 blend shape features are extracted^21,22^. With the landmarks around the eye, the eye aspect ratio (EAR; Fig. 1) can be calculated for both eyes over all frames of the video^23^. EAR describes the ratio between the vertical and horizontal distance between the landmarks, resulting in a detailed behavior approximation of the upper and lower eyelids. Hence, the EAR is characterizing the eye openness in each frame and invariant to the distance of the eye to the camera. The EAR is getting close to zero when closing the eye in a healthy person. The lower EAR, the better is the eye closure function. Furthermore, the blinks for both eyes were detected and counted.

### Statistics

All statistical analyses were performed using IBM SPSS Statistics 25 (Chicago, IL). The results had exploratory character as no measurements with the tool box had been performed in patients with facial palsy before. Hence, no data were available to determine a concrete assumption on the blinking in patients compared to healthy probands. Nevertheless, we performed a power analysis to get an idea of a sufficient sample size. Primary outcome measure was blinking per 20 min. Normal average spontaneous blinking is about 15/min, i.e. 300/20 min. Pooled for patients (acute and chronic palsy), we assumed a reduction to 100/20 min. Further, we assumed the same standard deviation of 200/20 min in probands and patients. Based on these assumptions, the power calculation revealed at a test level of alpha = 0.05, in each group (probands and patients) N = 23 participants had to be analyzed (two-sided independent samples) with a power of 95%. Therefore, we decided to include N = 30 patients and N = 30 matched healthy probands into the study.

Nominal and ordinal data are presented as absolute values and relative values in percentage. The results of the metric parameters are presented as means ± standard deviation (SD), median and range, if not otherwise indicated. In order to proof the hypothesis that spontaneous blinking parameters were impaired in patients compared to healthy controls, one factor analysis of variance (ANOVA) with post-hoc Bonferroni correction for multiple testing was used for all independent blinking parameters of all three subgroups (acute palsy, synkinesis, healthy). As the healthy controls had no paretic side, it was necessary to define which side (left/right) should be compared to the paretic side in the patients and which side to the contralateral side. The results for all blinking parameters for left and right side were not different in the controls (see “Results”). Therefore, it was determined to compare the paretic side of patients to the left side of controls. The contralateral side of patients was compared to the right side of controls. The Wilcoxon test was used to compare dependent parameters between two subgroups (paretic versus contralateral side). In order to proof the hypothesis that impaired blinking correlated to impaired quality of life, Spearman’s rho was used to perform the correlations analysis between the blinking parameters and the results of the PROMs. As the correlation analyses had exploratory character, no correction for multiple test was performed. P values < 0.05 were considered significant.

### Ethics statement

Written informed consent was obtained from all participants. Informed consent has also been obtained to publish the facial images in a publication. The ethics committee of the Jena University Hospital approved the study (No. 2019-1539).

## Results

### Characteristics and facial-specific quality of the life of the of patients and the healthy controls

Most of the patients with acute facial palsy had an idiopathic facial palsy (83.3%). Infection (37.5%) and trauma/tumor (33.3%) were to most frequent etiologies in the patients with facial synkinesis. The House-Brackmann grading varied from grade II to grade VI in the patients with acute palsy and from grade II to grade V in the patients with facial synkinesis. The median Sunnybrook Composite Score for the patients with acute facial palsy and for patients with facial synkinesis was 36.5 and 67, respectively. The median Sunnybrook Synkinesis Score of the patients with facial synkinesis was 6.5. More details are given in Table 1. The results of the facial-specific quality of life assessments are shown in Table 2. The one-way ANOVAs revealed that there was a statistically significant difference in all quality of life scores between at least two groups (Supplemental Table 1). As expected, the FaCE and FDI parameters were normal in the healthy controls. The FaCE and FDI domains were all decreased in patients with facial palsy. Nearly all parameters in patients were lower than in the healthy control group (mostly p < 0.001). Most values were not significantly different between patients with acute facial palsy and facial synkinesis (all p > 0.05) with exception of the FaCE subdomain Facial Comfort: Facial Comfort was significantly lower in the patients with facial synkinesis (p < 0.001).

**Table 1:** Characteristics of the healthy control group and the two patients groups.

Parameter	Healthy probands		Acute facial palsy		Facial synkinesis	
	N	%	N	%	N	%
All	30	100	6	100	24	100
Gender						
Female	20	66.7	4	66.7	16	66.7
Male	10	33.3	2	33.3	8	33.3
Etiology						
Idiopathic			5	83.3	7	29.2
Infectious			1	16.7	9	37.5
Trauma/tumor			0	0	8	33.3
Paretic side						
Left			2	33.3	15	62.5
Right			4	66.7	9	37.5
Complete bilateral eye closure						
Yes	30	100	2	33.3	19	79.2
No	0	0	4	66.7	5	20.8
Impaired vision						
No	30	100	1	16.7	7	29.2
Myopia	0	0	1	16.7	6	25
Hyperopia	0	0	2	33.3	7	29.2
Both	0	0	2	33.3	4	16.7
Handiness						
Left	3	10	0	0	0	0
Right	27	90	6	100	24	100
House-Brackmann scale						
Grade I	30	100				
Grade II			1	16.7	9	37.5
Grade III			2	33.3	10	41.7
Grade IV			1	16.7	3	12.5
Grade V			0	0	2	8.3
Grade VI			2	33.3	0	0

Parameter	M ± SD	Median; range	M ± SD	Median; range	M ± SD	Median; range
Age, years	57.5 ± 13.7	57; 22–83	58.1 ± 16.2	65; 29–72	57.2 ± 15.4	59; 22–81
Duration of palsy, months			0	0	46.8 ± 53.5	27.5; 238
Duration of palsy, days			3.2 ± 1.5	3.5, 1–5		
Sunnybrook grading						
Resting symmetry			6.7 ± 7.5	5; 0–15	4.6 ± 5.7	0; 0–15
Voluntary movement			48.7 ± 29.1	80; 24–100	74.3 ± 17.6	44; 20–92
Synkinesis			0	0	5.8 ± 3.4	6.5; 0–13
Composite score			42.0 ± 31.9	36.5; 9–92	65.0 ± 18.6	67; 22–93

M, mean; SD, standard deviation.

**Table 2:** Facial-specific quality of life of the healthy control group, acute facial palsy and postparalytic synkinesis group.

Parameter	Healthy probands	Acute facial palsy	Facial synkinesis	Healthy vs acute palsy	Healthy vs synkinesis	Acute vs synkinesis
	M ± SD	M ± SD	M ± SD	p*	p*	p*
FaCE						
Facial movement	100 ± 0	43.1 ± 40.3	42.7 ± 23.4	** < 0.001**	** < 0.001**	1.000
Facial comfort	99.4 ± 3	87.5 ± 13.7	41.3 ± 26.3	0.386	** < 0.001**	** < 0.001**
Oral function	100 ± 0	56.3 ± 36.9	60.9 ± 28.1	** < 0.001**	** < 0.001**	1.000
Eye comfort	93.6 ± 17.6	39.6 ± 29	43.2 ± 36.5	** < 0.001**	** < 0.001**	1.000
Lacrimal comfort	100 ± 0	50 ± 41.8	53.1 ± 32.4	** < 0.001**	** < 0.001**	1.000
Social function	100 ± 0	75 ± 19.4	58.3 ± 32	**0.032**	** < 0.001**	0.268
Total score	99.1 ± 2.4	62.2 ± 16.5	49.8 ± 21.7	** < 0.001**	** < 0.001**	0.210
FDI						
Physical function	99.2 ± 2.3	58.3 ± 15.4	62.1 ± 16.5	** < 0.001**	** < 0.001**	1.000
Social function	84.5 ± 15.4	74.7 ± 14.9	58.7 ± 24.7	0.798	** < 0.001**	0.239
Total score	91.9 ± 7.8	66.5 ± 11.5	60.4 ± 18.4	** < 0.001**	** < 0.001**	0.958

FaCE, Facial Clinimetric Evaluation; FDI, Facial Disability Index; M, mean; SD, standard deviation.

Significant values are in [bold].

*ANOVA, post-hoc test; additional data is given in Supplemental Table 1.

### Comparison of blinking on the paretic and the contralateral side

**Figure 2:**
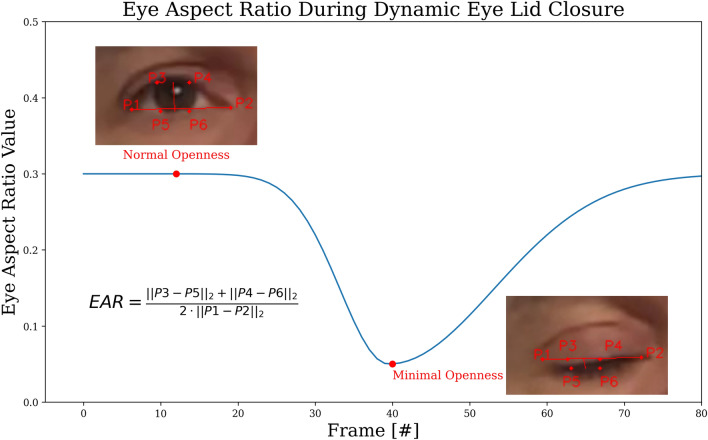
Automated blinking analysis over 20 min for the patients with acute facial palsy (blue line), patients with facial synkinesis (red line), and healthy probands (grey line). (**A**, **B)** Average number of blinks per minute (mean ± standard error of the mean). (**C**, **D)** Average blink duration in ms (mean ± standard error of the mean). (**A**, **C)** Paretic side of the patients, left side of the healthy probands. (**B**, **D)** Contralateral side of the patients, right side of the healthy probands.

All blinking analysis parameters for both facial sides are listed for the in Table 3. There was no side difference in the healthy controls (all p > 0.05). In patients with acute facial palsy, the ratio of the length of the palpebral fissure height (i.e. the highest to lowest point of the palpebral fissure) on the diseased side to the contralateral side was 96 ± 7%. The average and maximum EAR were not different (p = 0.248 and p = 0.345, respectively). The minimal EAR was greater on the paretic side, i.e. the ability to close the eye was lower (p = 0.028). The number of blink in 20 min and therefore also the blinking frequency was reduced on the paretic side (both p = 0.027). The same was seen for the subset of blinks with complete eye closure (both p = 0.028). The average duration of the blinks showed no side difference (p = 0.180). In patients with facial synkinesis, nearly all parameters were changed on the paretic side. The ratio of the length of the palpebral fissure height was 88 ± 18%. There was a trend to lower ratio compared to patients with acute facial palsy (p = 0.080). The average and the maximum EAR were reduced (p = 0.005 and p = 0.006, respectively), whereas the minimum EAR was larger than on the contralateral side (p = 0.004). The maximal EAR on the contralateral side in patients with facial synkinesis was reached the highest values from all sides. It might be that these patients actively make their contralateral eye more open to cope with the synkinesis on the paretic side. Blinking frequency was reduced on the post-paralytic synkinetic side (p < 0.001). The same was seen when only analyzing the blinks with complete eye closure (p < 0.001). There was a non-significant trend of a longer average duration of each blink on the synkinetic side (p = 0.080). The time course of the average number of blinks and the average duration of each blink during the 20 min observation time is shown in Fig. 2. The time course of only the number of blinks with complete eye closure is shown in Supplement Fig. 2. The number of blinks varied from minute to minute in healthy probands and in the patients. In contrast, the duration of each blink was relatively constant except for patients with acute palsy. Here, the duration of the blinks varied considerably.

**Table 3:** Automated blinking analysis of the healthy control group, acute facial palsy and postparalytic synkinesis group comparing the paralytic with the contralateral side.

Parameter	Healthy probands			Acute facial palsy				Facial synkinesis		
	Left	Right	p	Palsy side	Contralat	p	Palsy side		Contralat. side	p
	M ± SD	M ± SD		M ± SD	M ± SD		M ± SD		M ± SD	
Eye aspect ratio*										
Average	0.29 ± 0.04	0.29 ± 0.04	0.673	0.24 ± 0.07	0.27 ± 0.09	0.249	0.30 ± 0.05		0.32 ± 0.04	**0.005**
Minimum	0.03 ± 0.03	0.03 ± 0.04	0.627	0.13 ± 0.01	0.01 ± 0.01	**0.028**	0.06 ± 0.06		0.02 ± 0.03	**0.004**
Maximum	0.39 ± 0.05	0.39 ± 0.04	0.797	0.45 ± 0.06	0.35 ± 0.07	0.345	0.46 ± 0.18		0.48 ± 0.18	**0.006**
Blinks**, all										
Number in 20 min	254.3 ± 212.4	255.4 ± 211.6	0.273	47.3 ± 162.6	244 ± 24	**0.027**	132.7 ± 134.1		222.9 ± 156.1	** < 0.001**
Frequency, min	12.7 ± 10.6	12.8 ± 10.6	0.273	2.3 ± 8.1	12.2 ± 1.2	**0.027**	6.6 ± 6.7		11.1 ± 7.8	** < 0.001**
Duration, ms	199.7 ± 42.8	199.9 ± 48.9	0.787	364 ± 168.3	234 ± 24	0.180	240.8 ± 58.3		218.1 ± 41.5	0.080
Blinks, complete eye closure										
Number in 20 min	148.6 ± 160.7	148.2 ± 155.5	0.307	10.8 ± 43.1	241 ± 28.3	**0.028**	33.5 ± 46		155.1 ± 147.8	** < 0.001**
Frequency, min	7.4 ± 8	7.4 ± 7.8	0.307	0.5 ± 2.2	12.1 ± 1.4	**0.028**	1.7 ± 2.3		7.8 ± 7.4	** < 0.001**

Significant values are in [bold].

*Ratio of height/width of the eye opening, average of the first 3 s of the blink-free interval.

**At least pupil covered.

### Comparison of blinking between heathy probands and both patient groups

The comparison of the paretic side in the two patient groups and the left side in the healthy probands is shown in Table 4. The one-way ANOVAs revealed that there was a statistically significant difference in most blinking parameters on the paretic side (left side in controls) between at least two groups (Supplemental Table 2). Hence, the objective to show that the used tool box allowed an automated and objective confirmation of the impaired blinking in patients was confirmed. Regarding the EAR, only the minimum EAR, i.e. best eye closure showed significant lower values (better closure) for healthy probands compared to patients with acute facial palsy (p < 0.001) and lower values for patients with synkinesis than for patients with acute palsy (p = 0.015). The absolute number and hence also the blinking frequency was lower in both patient groups (acute palsy and patients with synkinesis) than in healthy probands (p = 0.034 and p = 0.034, respectively). The parameter were not different between the patient groups (p = 0.893). The same was seen when only the blinks with complete eye closure were examined. The average duration of the blinks was longer in both patients groups (p < 0.001 and p = 0.027, respectively), and also significantly longer in patients with acute palsy compared to the patients with facial synkinesis (p = 0.011).

**Table 4:** Blinking on the paretic/left* side, comparison of the healthy control group, acute facial palsy and postparalytic synkinesis group.

Parameter	Healthy probands	Acute facial palsy	Facial synkinesis	Healthy vs acute palsy	Healthy vs synkinesis	Acute vs synkinesis
	M ± SD	M ± SD	M ± SD	p*	p*	p*
Eye aspect ratio**						
Average	0.29 ± 0.04	0.24 ± 0.07	0.30 ± 0.05	0.178	1.000	0.084
Minimum	0.03 ± 0.03	0.13 ± 0.01	0.06 ± 0.06	** < 0.001**	0.110	**0.015**
Maximum	0.39 ± 0.05	0.45 ± 0.06	0.46 ± 0.18	1.000	0.322	1.000
Blinks**, all						
Number in 20 min	254.3 ± 212.4	47.3 ± 162.6	132.7 ± 134.1	**0.034**	**0.043**	0.893
Frequency, min	12.7 ± 10.6	2.3 ± 8.1	6.6 ± 6.7	**0.034**	**0.043**	0.893
Duration, ms	199.7 ± 42.8	364 ± 168.3	240.8 ± 58.3	** < 0.001**	**0.027**	**0.011**
Blinks, complete eye closure						
Number in 20 min	148.6 ± 160.7	10.8 ± 43.1	33.5 ± 45.5	**0.036**	**0.002**	1.000
Frequency, min	7.4 ± 8	0.5 ± 2.2	1.7 ± 2.3	**0.036**	**0.002**	1.000

Significant values are in [bold].

*ANOVA, post-hoc test, additional data is given in Supplemental Table 2; **left side in healthy controls.

The comparison of the contralateral side in the two patient groups and the left side in the healthy probands is shown in Table 5. The one-way ANOVAs revealed that there was no statistically significant difference in most blinking parameters on the contralateral side (right side in controls) between at least two groups (Supplemental Table 3). A difference between at least two groups was seen only for the average EAR on the contralateral side. Most parameters were not different between the three groups (all p > 0.05). Only the average EAR was higher (i.e. the eyes were more open) in the group of patients with synkinesis than in patients with acute palsy (p = 0.029) and also than in healthy probands (p = 0.017).

**Table 5:** Blinking on the contralateral/right* side, comparison of the healthy control group, acute facial palsy and postparalytic synkinesis group.

Parameter	Healthy probands	Acute facial palsy	Facial synkinesis	Healthy vs acute palsy	Healthy vs synkinesis	Acute vs synkinesis
	M ± SD	M ± SD	M ± SD	p*	p*	p*
Eye aspect ratio**						
Average	0.29 ± 0.04	0.27 ± 0.09	0.32 ± 0.04	1.000	**0.017**	**0.029**
Minimum	0.03 ± 0.04	0.01 ± 0.01	0.02 ± 0.03	0.519	0.951	1.000
Maximum	0.39 ± 0.04	0.35 ± 0.07	0.48 ± 0.18	0.850	0.071	1.000
Blinks**, all						
Number in 20 min	255.4 ± 211.6	244 ± 24	222.9 ± 156.1	1.000	1.000	1.000
Frequency, min	12.8 ± 10.6	12.2 ± 1.2	11.1 ± 7.8	1.000	1.000	1.000
Duration, ms	199.9 ± 48.9	234 ± 24	218.1 ± 41.5	0.316	0.349	1.000
Blinks, complete eye closure						
Number in 20 min	148.2 ± 155.5	241 ± 28.3	155.1 ± 147.8	1.000	1.000	1.000
Frequency, min	7.4 ± 7.8	12.1 ± 1.4	7.8 ± 7.4	1.000	1.000	1.000

Significant values are in [bold].

*ANOVA, post-hoc test, additional data is given in Supplemental Table 3; **right side in healthy controls.

### Correlation analysis between the blinking parameters and quality of life

An overview about the correlation analyses is given in Table 6. No correlations were seen for almost all blinking parameter and PROM values (all p > 0.05). Only in patients with acute facial palsy a better FaCE Eye Comfort was correlated to a higher blinking frequency (rho = 0.845; p = 0.034). Hence, the hypothesis that impaired spontaneous blinking is correlated to impaired quality was not confirmed.

**Table 6:** Correlation between blinking parameters on the paretic side//left* side and facial-specific quality of life.

	EAR; average	EAR; minimum	EAR; maximum	Blinks, number	Blinks, frequency	Blinks, duration	Blinks, eye closed, number	Blinks, eye closed, frequency
Healthy probands								
FaCE Eye Comfort	rho = − 0.145 p = 0.445	rho = 0.026 p = 0.890	rho = − 0.294 p = 0.115	rho = − 0.242 p = 0.198	rho = − 0.242 p = 0.198	rho = 0.192 p = 0.309	rho = 0.116 p = 0.542	rho = 0.116 p = 0.542
FaCE Total Score	rho = − 0.177 p = 0.349	rho = 0.026 p = 0.890	rho = − 0.302 p = 0.105	rho = − 0.314 p = 0.092	rho = − 0.314 p = 0.092	rho = 0.084 p = 0.66	rho = 0.085 p = 0.656	rho = 0.085 p = 0.656
FDI item 4*	rho = − 0.156 p = 0.409	rho = 0.042 p = 0.825	rho = − 0.284 p = 0.128	rho = − 0.239 p = 0.203	rho = − 0.239 p = 0.203	rho = 0.197 p = 0.297	rho = 0.109 p = 0.565	rho = 0.109 p = 0.565
FDI Total Score	rho = − 0.058 p = 0.762	rho = 0.124 p = 0.513	rho = 0.086 p = 0.65	rho = 0.033 p = 0.863	rho = 0.033 p = 0.863	rho = 0.300 p = 0.108	rho = − 0.229 p = 0.223	rho = − 0.229 p = 0.223
Patients with acute facial palsy								
FaCE Eye Comfort	rho = 0.029 p = 0.957	rho = − 0.600 p = 0.208	rho = 0.029 p = 0.957	rho = 0.845 **p = 0.034**	rho = 0.845 **p = 0.034**	rho = − 1 p = NA	rho = 0.845 p = 0.034	rho = 0.845 p = 0.034
FaCE Total Score	rho = 0.2 p = 0.704	rho = − 0.314 p = 0.544	rho = 0.200 p = 0.704	rho = 0.676 p = 0.140	rho = 0.676 p = 0.140	rho = − 1 p = NA	rho = 0.676 p = 0.140	rho = 0.676 p = 0.140
FDI item 4*	rho = 0.062 p = 0.908	rho = − 0.679 p = 0.138	rho = − 0.463 p = 0.355	rho = 0.876 p = 0.022	rho = 0.876 p = 0.022	rho = 1 p = NA	rho = 0.876 p = 0.022	rho = 0.876 p = 0.022
FDI Total Score	rho = 0.486 p = 0.329	rho = 0.314 p = 0.544	rho = 0.086 p = 0.872	rho = 0.101 p = 0.848	rho = 0.101 p = 0.848	rho = − 1 p = NA	rho = 0.101 p = 0.848	rho = 0.101 p = 0.848
Patients with facial synkinesis								
FaCE Eye Comfort	rho = 0.084 p = 0.695	rho = 0.263 p = 0.215	rho = − 0.115 p = 0.592	rho = 0.078 p = 0.716	rho = 0.078 p = 0.716	rho = − 0.130 p = 0.555	rho = 0.204 p = 0.340	rho = 0.204 p = 0.340
FaCE Total Score	rho = − 0.054 p = 0.804	rho = 0.285 p = 0.178	rho = 0.011 p = 0.959	rho = 0.081 p = 0.705	rho = 0.081 p = 0.705	rho = − 0.169 p = 0.441	rho = 0.181 p = 0.396	rho = 0.181 p = 0.396
FDI item 4*	rho = − 0.072 p = 0.738	rho = 0.107 p = 0.617	rho = − 0.312 p = 0.138	rho = 0.057 p = 0.790	rho = 0.057 p = 0.790	rho = 0.142 p = 0.518	rho = 0.183 p = 0.393	rho = 0.183 p = 0.393
FDI Total Score	rho = − 0.125 p = 0.561	rho = 0.240 p = 0.259	rho = 0.273 p = 0.197	rho = − 0.139 p = 0.517	rho = − 0.139 p = 0.517	rho = − 0.027 p = 0.904	rho = − 0.019 p = 0.928	rho = − 0.019 p = 0.928

Significant values are in [bold].

EAR, eye aspect ratio.

*Left side in healthy controls; **this FDI question addresses directly the eye function: “How much difficulty did you have with your eyes tearing excessively or becoming dry?”.

## Discussion

The main objective, to establish the tool box for use in clinical routine and to show the feasibility to measure objectively spontaneous blinking parameters, has been achieved by the presented study. Furthermore, the hypothesis, that impaired blinking in patients with facial palsy could be measured automatically, could be confirmed. In contrast, the hypothesis that the blinking impairment correlates with impaired quality of life could not be confirmed.

Blinking is a dynamic facial nerve related facial function important for corneal protection and optimal vision. Spontaneous blinking consists of a stereotypic rapid downward movement of the upper eyelid and a subsequent upward movement completing the blink. This is not the same as the eye closure typically performed during facial function assessment with facial grading systems. There is no established facial grading tool for routine use estimating eye blinking function^24^. Terzis and Bruno suggested in 2002 a subjective 5-stage scoring system for grading of blinks^25^. However, this system was never used again by others.

The present study confirms that blinking is reduced on the paretic side in patients with acute facial palsy. Moreover, impaired blinking could also be confirmed for patients with facial synkinesis. The use of an automated image analysis tool allowed an easy, not time-consuming, and reliable quantification of the blinking frequency and of the blinking duration. The later was significantly prolonged in patients with facial palsy. The automated method also allowed a precise quantification of the eye opening area or the degree of eye closure by calculation of the EAR. The present results show that these parameters are also not adequately covered by facial-specific PROMs like the FDI or the FaCE. We could not reveal any relevant correlation between the blinking parameters and these PROMs. The reason might be that neither the FDI nor the FACE ask directly for blinking problems. We are not aware of any PROM directly addressing blinking. Even the National Eye Institute Visual Function Questionnaire (NEI VFQ-25), a frequently used PROM to measure vision related quality of life does not ask for blinking^26^.

The standard for eyelid movement and blinking analyses uses electrooculography, or EMG recordings from the orbicularis oculi muscle in combination with magnetic coils or a gyroscope to measure the vertical upper eyelid movement^2,27,28^. Such approaches allow a detailed analysis of blink kinematics but needs neurophysiological expertise and are too complex for fast use in a clinical routine setting compared to video recordings. In a next study, we will analyze if we even can use videos that the patients recorded themselves at home. Terzis and Bruno suggested the measurement of a blink percentage score^25^. They measured manually with a metric ruler on videos frame by frame the interpalpebral distance at the midpupillary line. This is not only too time-consuming for routine use, it is also not reliable due to non-standardized measurement conditions. Already in 2005 Schellini et al.^29^ used 3-min videos with a 30 s frame rate and a commercial movie software to measure manually in the videos the eyelid opening and closing time for normal spontaneous blinks. Coulson et al.^30^ also used videos (with a 25 s frame rate) to analyze manually the bilateral conjugacy of movement initiation of the eyelid movement in patients with unilateral chronic facial palsy. They showed that the initiation of movement of the paretic and non-paretic eyelids was synchronous, but markedly delayed relative to healthy probands. Synchronicity of the blinking was not yet analyzed by us, but should be implemented in the further analyses. Blepharokymography is also a video-based blinking analysis tool already used in patients with Bell’s palsy to study voluntary blinks but still was a semiautomatic procedure^31^. Osaki et al.^32^ used a high-speed video system and automated analysis to analyze blink activity in patients with hemifacial spasms but needed the placement of a light-emitting diode on the pretarsal region of the upper eyelid. Modern artificial intelligence based video-based automated blink detection tools were mainly used so far in fields outside medicine. A large field is for instance the market for car driver drowsiness detection tools^10^. Recently, a first program based on a deep learning model using convolutional neural network architecture was presented to automatically measure margin reflex distances of the eyes^13^. This allowed the measurement of the ocular surface area exposure in patients with facial palsy. The inclusion of artificial intelligence was the important step, also for the present study, to overcome older major drawbacks of video-based analysis. This methodology is able to handle important factors influencing the results like movements of the head, variation of the distance to the camera, changes of the light, variability of eyelid skin and color of the iris^28^.

The variability in the methodology has to be taken into account when comparing our results to the literature. On average, about 12–13 blinks per minute with a blink duration of about 200 ms were counted in healthy probands. About 7 blinks per minute, i.e. about half of all blinks produced a complete eye closure in healthy probands. In the literature using manual methods for counting, the spontaneous blink rate in adults between 50 and 70 years varies between 11 and 22 blinks per minute, i.e. the presented results fall into this range (see Fig. 5 in:^27^). Due to classical EMG studies, the duration of the orbicularis oculi activation during a normal spontaneous blink is about 280–300 ms^33^. This fits well to the present results as EMG activity is seen before the movement occurs^33,34^. The blink frequency was decreased and the blink duration was increased both in patients with acute facial palsy and in patients with facial synkinesis. The latter is shown for the first time. Furthermore, it seems that the patients with acute facial palsy try to compensate the disturbed ipsilateral blinking with a longer blink duration on the contralateral side. It would therefore be interesting to develop a training program for voluntary blinking and to see whether this improves patients’ quality of life.

The sample size was too small to be able to give a definitive answer here. Studies on blinking in patients with facial palsy were so far focused on patients with acute palsy. We clearly show that disturbed blinking is also an important factor for patients with facial synkinesis. The EAR as a measure of the eye openness or closeness is a very robust parameter in automated video analysis^23^, but was not yet used as parameter in patients with facial palsy. The minimal EAR as measure of minimal openness (maximal closeness) of the eye again was disturbed not only in patients with acute facial palsy but also in patients with facial synkinesis. Schulz et al.^13^ used the margin reflex distance (MRD) as parameter of the eye openness. They showed an increased MRD in patients with acute facial palsy going back to normal after recovery. Patients with facial synkinesis were not evaluated.

The present study has limitations. The group of patients with acute facial palsy in this first study using the JeFaPaTo was very small. A larger group will obtain more robust but probably not other results. Spontaneous blinks were analyzed. The other two types, voluntary and reflex blinks were not yet investigated^2^. Furthermore, spontaneous blinking was only analyzed at rest. A patient with synkinesis who is talking or smiling may close his eyes unexpectedly. This is analyzed in an ongoing study. Then, blinking activity during daily activity depends on several internal and external factors, including age, ocular surface status, level of mental activity, changes in visual processing or attention^27,35^. It would be worthwhile to analyze if spontaneous blinking is differently changed during attention and social communications tasks in patients with facial palsy compared to healthy probands^35^.

Some classical blink parameter like blink peak velocity and amplitude (that are also disturbed in the patients) are not yet implemented in the tool^36,37^. Furthermore, asymmetry of blinking might be perceived as disturbing for the patients^34^. This parameter should be implemented in the software, too. A 6-year old smart phone allowing videos with 240 frames per second was used. Nowadays, many smartphone allow such a frame rate and are ubiquitously available. Nevertheless, JeFaPaTo would also allow analysis of videos with lower frame rate. The other way round, JeFaPaTo would also allow to upload data from ultrahigh-speed cameras like they are used for basic research questions related to eye blinking^38^.

Beyond the addition of further blink parameters to the software it will be worthwhile to use the tool also for other oculofacial disease and conditions with disturbed eye blinking. Typical examples are hemifacial spasm, blepharospasm, Grave’s disease, and Parkinson’s disease, but also research conditions like sleep deprivation or settings with alternating attention^13,27,32,39^.

## Conclusions

Automated, objective and fast analysis of spontaneous eye blinking is feasible in patients with facial palsy and postparalytic facial syndrome with synkinesis. Blinking is decreased and blink duration is prolonged not only in patients with acute facial palsy but also in patients with facial synkinesis. Although the number of blinks with complete eye closure remains decreased on patients with facial synkinesis compared to healthy controls, the minimal openness of the eye surface returns nearly back to normal. All aspects of blinking seem not to be covered by typical facial-specific PROMs, as the FDI and the FaCE do not correlate to the results of the blinking analyses. Automated blinking analysis should be used in routine grading of facial palsy, in clinical studies, and to compare groups of patients from different institutions.

### Supplementary Information

Supplementary Figures.

Supplementary Tables.

## Data Availability

The original contributions presented in the study are included in the article and in the supplementary material. Further inquiries can be directed to the corresponding author.
