# Supplementary material for: Automated analysis of spontaneous eye blinking in patients with acute facial palsy or facial synkinesis
Source: Sci Rep. 2024 Jul 31;14:17726. doi: 10.1038/s41598-024-68707-x (PMC11292012; doi:10.1038/s41598-024-68707-x)

**Automated analysis of spontaneous eye blinking in patients with acute facial palsy or facial synkinesis**

Lukas Schuhmann^1^, Tim Büchner^2^, Martin Heinrich^1,3,4^, Gerd Fabian Volk^1,3,4^, Joachim Denzler^2^, Orlando Guntinas-Lichius^1,3,4*^

**Supplement Figures**

**Supplement Fig. 1.** Setting for the smartphone video recordings of the spontaneous eye blinks. The participants were sitting in front of a computer screen watching an animal movie over 20 min. The smartphone was installed below the computer screen (arrow). Distance between screen and eyes: 50 cm. Distance between camera and eyes: 45 cm.

**Supplement Fig. 2.** Automated blinking analysis over 20 minutes for the patients with acute facial palsy (blue line), patients with facial synkinesis (red line), and healthy probands (grey line). Shown is only the number of blinks per minute (mean±standard error of the mean) for blinks with complete eye closure. **A:** paretic side of the patients, left side of the healthy probands. **B:** contralateral side of the patients, right side of the healthy probands. Shown

**Supplement Figure 1**

**
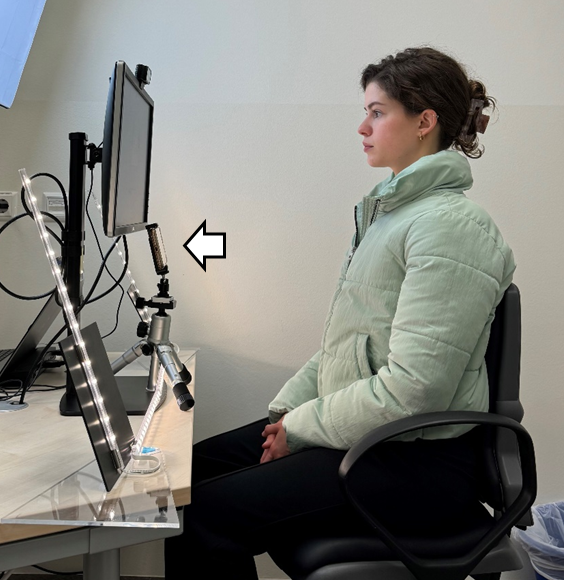
**

**Supplement Figure 2**


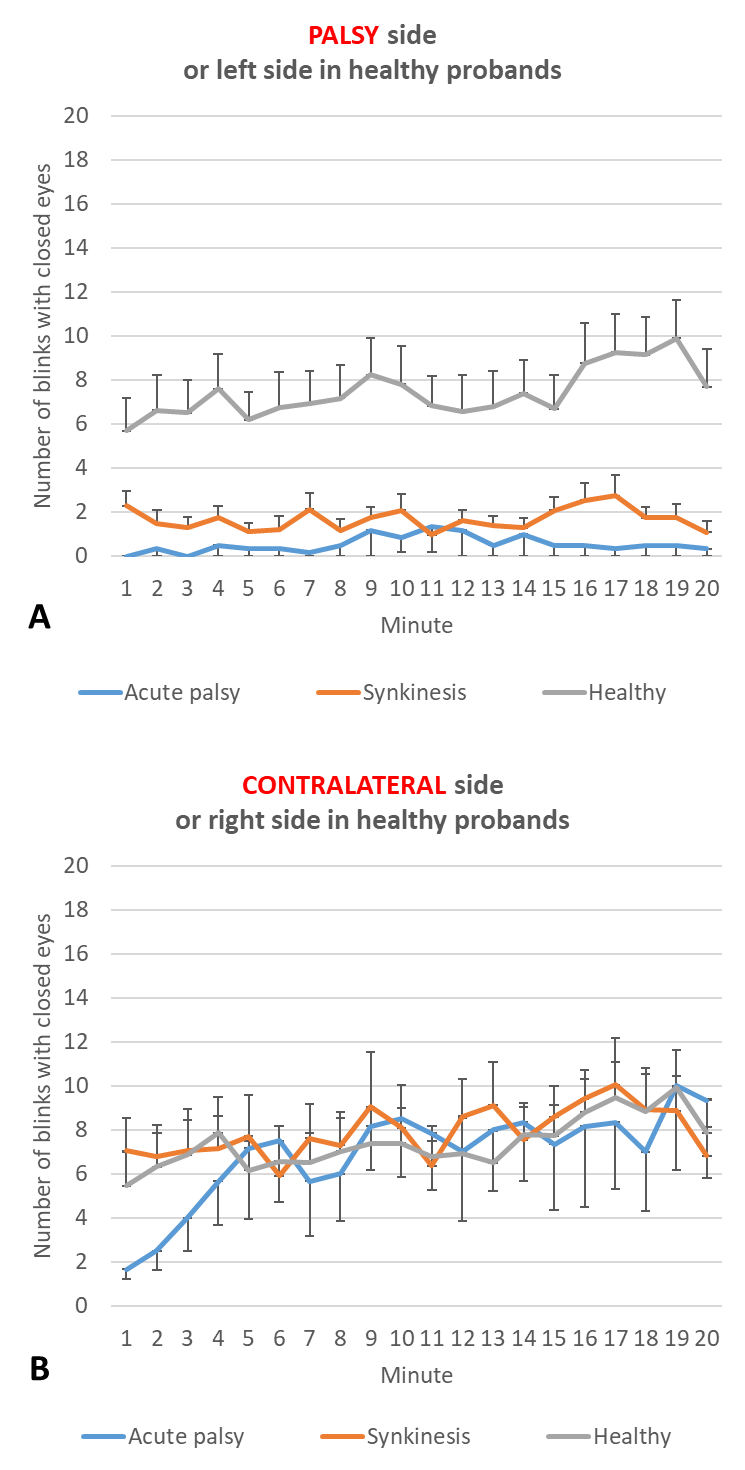

Supplement: Supplementary file 1 — Supplementary Figures. [file 41598_2024_68707_MOESM1_ESM.docx]
