# Supplementary material for: Automated analysis of spontaneous eye blinking in patients with acute facial palsy or facial synkinesis
Source: Sci Rep. 2024 Jul 31;14:17726. doi: 10.1038/s41598-024-68707-x (PMC11292012; doi:10.1038/s41598-024-68707-x)
Supplement: Supplementary file 2 — Supplementary Tables. [file 41598_2024_68707_MOESM2_ESM.docx]

**Automated analysis of spontaneous eye blinking in patients with acute facial palsy or facial synkinesis**

Lukas Schuhmann^1^, Tim Büchner^2^, Martin Heinrich^1,3,4^, Gerd Fabian Volk^1,3,4^, Joachim Denzler^2^, Orlando Guntinas-Lichius^1,3,4*^

**3 Supplemental Tables**

**Supplemental Table 1**

| **Supplemental Table 1.** Additional data for the one-way ANOVA comparing the facial specific quality of life between the healthy control group, acute facial palsy and postparalytic synkinesis group. | | | | | | |
| --- | --- | --- | --- | --- | --- | --- |
|  |  | Sum of squares | Df | Mean square | F | p |
| **FaCE** |  |  |  |  |  |  |
| Facial Movement | Between groups | 49116.319 | 2 | 24558.160 | 67.765 | **<0.001** |
|  | Within groups | 20656.829 | 57 | 362.401 |  |  |
|  | Total | 69773.148 | 59 |  |  |  |
| Facial Comfort | Between groups | 46088.542 | 2 | 23044.271 | 76.788 | **<0.001** |
|  | Within groups | 17105.903 | 57 | 300.104 |  |  |
|  | Total | 63194.444 | 59 |  |  |  |
| Oral Function | Between groups | 24105.469 | 2 | 12052.734 | 27.459 | **<0.001** |
|  | Within groups | 25019.531 | 57 | 438.939 |  |  |
|  | Total | 49125.000 | 59 |  |  |  |
| Eye Comfort | Between groups | 39462.240 | 2 | 19731.120 | 25.680 | **<0.001** |
|  | Within groups | 43795.573 | 57 | 768.343 |  |  |
|  | Total | 83257.813 | 59 |  |  |  |
| Lacrimal Comfort | Between groups | 33890.625 | 2 | 16945.313 | 29.367 | **<0.001** |
|  | Within groups | 32890.625 | 57 | 577.029 |  |  |
|  | Total | 66781.250 | 59 |  |  |  |
| Social Function | Between groups | 23375.000 | 2 | 11687.500 | 26.184 | **<0.001** |
|  | Within groups | 25442.708 | 57 | 446.363 |  |  |
|  | Total | 48817.708 | 59 |  |  |  |
| Total Score | Between groups | 33562.273 | 2 | 16781.136 | 77.251 | **<0.001** |
|  | Within groups | 12382.019 | 57 | 217.228 |  |  |
|  | Total | 45944.291 | 59 |  |  |  |
| **FDI** |  |  |  |  |  |  |
| Physical Function | Between groups | 21537.917 | 2 | 10768.958 | 80.945 | **<0.001** |
|  | Within groups | 7583.333 | 57 | 133.041 |  |  |
|  | Total | 29121.250 | 59 |  |  |  |
| Social Function | Between groups | 8935.467 | 2 | 4467.733 | 11.583 | **<0.001** |
|  | Within groups | 21986.133 | 57 | 385.722 |  |  |
|  | Total | 30921.600 | 59 |  |  |  |
| Total Score | Between groups | 13906.013 | 2 | 6953.006 | 38.948 | **<0.001** |
|  | Within groups | 10175.700 | 57 | 178.521 |  |  |
|  | Total | 24081.713 | 59 |  |  |  |

FaCE = Facial Clinimetric Evaluation; FDI = Facial Disability Index

**Supplemental Table 2**

| **Supplemental Table 2.** Additional data for the one-way ANOVA comparing the blinking on the paretic/left side*, comparison of the healthy control group, acute facial palsy and postparalytic synkinesis group. | | | | | | |
| --- | --- | --- | --- | --- | --- | --- |
|  |  | Sum of squares | Df | Mean square | F | p |
| **Eye Aspect Ratio** |  |  |  |  |  |  |
| Average | Between groups | 0.011 | 2 | 0.005 | 2.553 | 0.087 |
|  | Within groups | 0.121 | 57 | 0.002 |  |  |
|  | Total | 0.132 | 59 |  |  |  |
| Minimum | Between groups | 0.048 | 2 | 0.024 | 9.671 | **<0.001** |
|  | Within groups | 0.141 | 57 | 0.002 |  |  |
|  | Total | 0.189 | 59 |  |  |  |
| Maximum | Between groups | 0.058 | 2 | 0.029 | 1.479 | 0.237 |
|  | Within groups | 1.121 | 57 | 0.020 |  |  |
|  | Total | 1.179 | 59 |  |  |  |
| **Blinks, all** |  |  |  |  |  |  |
| Number in 20 min | Between groups | 325197.350 | 2 | 162598.675 | 5.219 | **0.008** |
|  | Within groups | 1775795.633 | 57 | 31154.309 |  |  |
|  | Total | 2100992.983 | 59 |  |  |  |
| Frequency, min | Between groups | 812.993 | 2 | 406.497 | 5.219 | **0.008** |
|  | Within groups | 4439.489 | 57 | 77.886 |  |  |
|  | Total | 5252.482 | 59 |  |  |  |
| Duration, ms | Between groups | 63356.505 | 2 | 31678.252 | 10.551 | **<0.001** |
|  | Within groups | 156119.604 | 52 | 3002.300 |  |  |
|  | Total | 219476.109 | 54 |  |  |  |
| **Blinks, complete eye closure** |  |  |  |  |  |  |
| Number in 20 min | Between groups | 217028.533 | 2 | 108514.267 | 7.734 | **0.001** |
|  | Within groups | 799796.200 | 57 | 14031.512 |  |  |
|  | Total | 1016824.733 | 59 |  |  |  |
| Frequency, min | Between groups | 542.571 | 2 | 271.286 | 7.734 | **0.001** |
|  | Within groups | 1999.491 | 57 | 35.079 |  |  |
|  | Total | 2542.062 | 59 |  |  |  |

*left side in normal controls.

**Supplemental Table 3**

| **Supplemental Table 3.** Additional data for the one-way ANOVA comparing the blinking the contralateral/right side*, comparison of the healthy control group, acute facial palsy and postparalytic synkinesis group. | | | | | | |
| --- | --- | --- | --- | --- | --- | --- |
|  |  | Sum of squares | Df | Mean square | F | p |
| **Eye Aspect Ratio** |  |  |  |  |  |  |
| Average | Between groups | 0.022 | 2 | 0.011 | 5.825 | **0.005** |
|  | Within groups | 0.109 | 57 | 0.002 |  |  |
|  | Total | 0.131 | 59 |  |  |  |
| Minimum | Between groups | 0.002 | 2 | 0.001 | 1.169 | 0.318 |
|  | Within groups | 0.054 | 57 | 0.001 |  |  |
|  | Total | 0.056 | 59 |  |  |  |
| Maximum | Between groups | 0.114 | 2 | 0.057 | 2.808 | 0.069 |
|  | Within groups | 1.157 | 57 | 0.020 |  |  |
|  | Total | 1.271 | 59 |  |  |  |
| **Blinks, all** |  |  |  |  |  |  |
| Number in 20 min | Between groups | 23358.850 | 2 | 11679.425 | 0.349 | 0.707 |
|  | Within groups | 1908880.133 | 57 | 33489.125 |  |  |
|  | Total | 1932238.983 | 59 |  |  |  |
| Frequency, min | Between groups | 58.397 | 2 | 29.199 | 0.349 | 0.707 |
|  | Within groups | 4772.200 | 57 | 83.723 |  |  |
|  | Total | 4830.597 | 59 |  |  |  |
| Duration, ms | Between groups | 8351.075 | 2 | 4175.538 | 2.064 | 0.136 |
|  | Within groups | 115290.658 | 57 | 2022.643 |  |  |
|  | Total | 123641.733 | 59 |  |  |  |
| **Blinks, complete eye closure** |  |  |  |  |  |  |
| Number in 20 min | Between groups | 2412.342 | 2 | 1206.171 | 0.055 | 0.946 |
|  | Within groups | 1240712.592 | 57 | 21766.888 |  |  |
|  | Total | 1243124.933 | 59 |  |  |  |
| Frequency, min | Between groups | 6.031 | 2 | 3.015 | 0.055 | 0.946 |
|  | Within groups | 3101.781 | 57 | 54.417 |  |  |
|  | Total | 3107.812 | 59 |  |  |  |

*right side in normal controls.
